# Menstrual cycle-dependent expression of tRNA-derived fragments in healthy human endometrium and uterine fluid-derived extracellular vesicles

**DOI:** 10.1186/s12864-026-12851-3

**Published:** 2026-04-14

**Authors:** Apostol Apostolov, Mladen Naydenov, Kristiyana Kambareva, Amruta D. S. Pathare, Merli Saare, Alberto Sola-Leyva, Andres Salumets, Vesselin Baev, Galina Yahubyan

**Affiliations:** 1 Celvia CC, Tartu, Estonia; 2 Department of Clinical Science, Division of Obstetrics and Gynecology, Intervention and Technology (CLINTEC), Karolinska Institute, Stockholm, Sweden; 3 Department of Gynecology and Reproductive Medicine, Karolinska University Hospital, Stockholm, Sweden; 4 Department of Biotechnology, Institute of Molecular and Cell Biology, University of Tartu, Tartu, Estonia; 5 Department of Human Anatomy and Physiology, Faculty of Biology, University of Plovdiv, Plovdiv, Bulgaria; 6 Department of Molecular Biology, University of Plovdiv, Plovdiv, Bulgaria; 7 Department of Obstetrics and Gynecology, Institute of Clinical Medicine, University of Tartu, Tartu, Estonia

**Keywords:** Endometrium, Receptivity, Menstrual cycle, tRNA, tRFs

## Abstract

The human endometrium undergoes dynamic cyclical remodeling across the menstrual cycle to establish receptivity for embryo implantation. While microRNAs are established regulators of endometrial function and embryo–maternal communication, the contribution of tRNA-derived fragments (tRFs) remains largely unexplored. In this study, we performed comprehensive small RNA sequencing of endometrial biopsy (EB) samples and paired uterine fluid–derived extracellular vesicles (UF-EVs) collected from healthy fertile women across consecutive menstrual cycle phases: mid-proliferative (MP), early secretory (ES), mid-secretory (MS), and late secretory (LS). Using the tRAX analytical framework, we characterized the landscape and compartmentalization of tRF populations. tRFs were detected from all 21 cytosolic tRNA isotypes in both EB and UF-EVs, displaying marked phase- and tissue-specific variation. In EB, tRFs were predominantly derived from tRNA-Lys and tRNA-Val, whereas UF-EVs exhibited greater isotype diversity, including substantial contributions from tRNA-Asp and tRNA-Gly. Fragment-type profiling revealed detection of 5′, 3′-, and internal tRF classes across all phases, supporting broad capture of diverse tRF subtypes. Notably, tRFs derived from tRNA-Val showed the most pronounced menstrual cycle–dependent regulation. Multiple 5′-tRF-Val species, including transcript- and isodecoder-specific fragments from tRNA-Val-CAC and tRNA-Val-AAC, were strongly upregulated during the ES and MS phases compared with the MP phase in both EB and UF-EVs. These coordinated increases suggest selective accumulation and extracellular export of tRNA-Val fragments during the secretory phase, potentially reflecting hormonally driven regulation of tRF biogenesis and vesicle-mediated signaling. Together, these findings provide the first comprehensive characterization of menstrual cycle–dependent tRF expression in the human endometrium and UF-EVs. Our results identify tRNA-Val–derived 5′-tRFs as prominent secretory-phase–associated small RNAs and support a potential role for tRFs as novel regulators and extracellular indicators of endometrial receptivity.

**Supplementary Information:**

The online version contains supplementary material available at 10.1186/s12864-026-12851-3.

## Introduction

The human endometrium undergoes cyclical remodeling driven by fluctuating ovarian hormones, coordinating proliferation, differentiation, and shedding essential for menstrual cyclicity and fertility. Successful embryo implantation depends on precise cellular dynamics within the endometrium during the window of implantation (WOI), including coordinated remodeling of epithelial, stromal, and immune compartments. Despite major insights from recent transcriptomics studies, the contributions of small non-coding RNAs especially their potential to regulate the mechanism of embryo-endometrium cross-talk remain emerging areas of active research [1–3].

Transfer RNA-derived fragments (tRFs) have recently emerged as an abundant and functionally diverse class of small non-coding RNAs, typically 14 to 30 nucleotides (nt) in length, generated by precise cleavage of precursor or mature tRNAs. Their classification varies across studies, reflecting evolving understanding of their biogenesis and functions. Broadly, tRNA-derived small RNAs are divided into longer tRNA halves (tiRNAs, 29–50 nt), produced under stress by cleavage at the anticodon loop [4, 5], and shorter tRFs (14–32 nt), generated by cleavage at various sites on precursor or mature tRNAs. Common tRF types include 5′-tRFs derived from the 5′-end of mature tRNAs, often cleaved near the D-loop; 3′-tRFs originating from the 3′-end, typically retaining the CCA tail [6, 7]; and tRF-1 fragments from the 3′-trailer of precursor tRNAs containing poly-U sequences [8]. Additional categories include internal tRFs (i-tRFs) derived from internal regions excluding the ends and less consistently defined tRF-2 fragments spanning the anticodon loop. Collectively, tRFs represent a complex and versatile group of small RNAs implicated in gene regulation, stress responses, and intercellular communication [9].

tRFs are increasingly recognized for their regulatory roles in reproductive tissues. In male reproduction, they are produced dynamically during sperm maturation, where they are selectively packaged into extracellular vesicles (EVs). This targeted trafficking modulates gene expression in sperm lineage and is essential for proper regulation of early embryonic development in mice [10, 11]. The selective delivery of these small RNAs via vesicles provides a mechanism for influencing both sperm function and epigenetic inheritance [10]. Notably, further studies have revealed that tRFs produced during spermatogenesis are subsequently delivered to the oocyte at fertilization, enabling tRFs to alter gene expression in the developing embryo and participate in epigenetic reprogramming and intergenerational signaling in mice [12]. In humans, seminal plasma EVs have been found to be enriched with specific tRF populations, suggesting that tRFs are selectively packaged and exported in EVs, and may participate in intercellular communication within the reproductive tract [13]. Additionally, human sperms have been shown to contain stable populations of tRFs that are delivered to the oocyte at fertilization, supporting their involvement in early embryonic gene regulation [14]. Together, these studies provide a systemic view that tRFs are integral components of the small RNA landscape in male reproductive tissues, where their abundance and composition are tightly regulated in a manner that reflects developmental stage, tissue type, and the physiological context. Moreover, these RNA molecules may play an important role in regulating the paternal effects on early embryogenesis via EV-mediated mechanisms.

In female reproduction, their function is just beginning to be explored. Dysregulated tRF expression has been observed in endometriosis, an estrogen-dependent disorder linked to infertility, with specific tRFs altered in ovarian endometriotic lesions and vaginal secretions. suggesting their potential roles in disease pathogenesis and use as non-invasive biomarkers [15, 16]. Beyond pathology, tRFs likely regulate gene expression, translation, and intercellular communication in reproductive tissues, often via EVs, thereby influencing endometrial remodeling and embryo-maternal signaling [17, 18]. Although mechanistic data in the female reproductive tract remain limited, the dynamic regulation and extracellular transport of tRFs, likely via EVs, underscore their emerging significance in reproductive health and diseases.

In this study, we performed comprehensive small RNA sequencing of endometrial biopsy (EB) samples and paired uterine fluid–derived extracellular vesicle (UF-EV) samples collected across the menstrual cycle from healthy fertile women. Our objective was to define the tRF repertoire in these compartments and to investigate how tRF abundance, fragment composition, and tRNA isotype origins vary across cycle phases. By providing the first phase- and compartment-resolved profiles of endometrial and uterine fluid EV–associated tRFs, our results highlight tRFs as dynamically regulated components of the small RNA landscape and suggest their potential involvement in endometrial remodeling.

## Materials and methods

### Ethical approval

The University of Tartu Research Ethics Committee in Estonia (No. 330 M-8) reviewed and approved the study in accordance with the Declaration of Helsinki, and written informed consent was obtained from all participants.

### Collection of endometrial biopsy and UF samples and isolation of EVs

A total of 50 endometrial biopsy and UF samples were collected across the menstrual cycle. The proliferative phase was determined according to menstrual cycle dating and mid-proliferative (MP) phase was determined as cycle day 7–8, while secretory phase stages were assigned relative to the luteinizing hormone (LH) surge, as detected using the BabyTime hLH urine cassette (Pharmanova, Belgrade, Serbia). Specifically, early secretory (ES) phase was defined as days LH + 2 to LH + 3, mid-secretory (MS) phase as LH + 7 to LH + 9, and late secretory (LS) phase as LH + 11 to LH + 12. Furthermore, molecular endometrial dating was performed using BeReady testing [19], in which the expression of 67 endometrial receptivity marker genes was assessed to validate the endometrial menstrual cycle phase.

Twenty-three paired EB-UF samples (*n* = 46) and 4 unpaired samples were collected. Specifically, 26 EB samples were collected from the MP (*n* = 5), ES (*n* = 7), MS (*n* = 7) and LS (*n* = 7) phases. Additionally, 24 UF samples from MP (*n* = 6), ЕS (*n* = 6), MS (*n* = 6) and LS (*n* = 6) phases were also collected. The participants were healthy, fertile women of reproductive age with at least one live-born child, who had abstained from the use of hormonal medications for a minimum of three months prior to participation, with a mean age of 32.1 ± 5.5 years and a mean BMI of 24.7 ± 3.1 kg/m² [20].

UF samples were obtained via transcervical lavage using 0.5 mL of phosphate-buffered saline and a sterile intrauterine insemination catheter (Cooper Surgical, Trumbull, CT, USA). Following UF aspiration, endometrial biopsy samples were collected using a Pipelle flexible suction catheter (Laboratoire CCD, Paris, France) and immediately preserved in HypoThermosol FRS Preservation Solution (Sigma, USA) for subsequent processing.

EVs were isolated from UF using miniPURE-EV SEC columns (HansaBioMed Life Sciences Tallinn, Estonia) as previously described [20]. Furthermore, the EVs were precipitated from the EV suspension with the evGAG (HansaBioMed Life Sciences), using a reagent-to-sample volume ratio of 1:1, according to the manufacturer’s instructions. Following isolation, EVs from each sample were immediately processed for RNA extraction to preserve EV integrity and avoid freeze–thaw related degradation.

Previously, this sample cohort was used for mRNA and small RNA sequencing. The UF-EVs used in this work have been extensively characterized in accordance with the MISEV guidelines [21] in our previously published article, including nanoparticle tracking analysis, western blotting, flow cytometry, and transmission electron microscopy [20]. The present work extends these analyses to tRFs within the same cohort.

### Small-RNA extraction library preparation and sequencing

RNA from EB and UF-EVs (particle number ranging from 1.90^10^ to 1.7 × 10^11^) was extracted with QIAzol (Qiagen, Germany) and miRNeasy micro kit (Qiagen, Hilden, Germany) by consecutively separating the large and small RNA fractions. The small RNA fraction was quantified with Qubit microRNA assay (Thermo Fisher Scientific). Small RNA libraries were generated using the NEXTflex Small RNA Library Preparation Kit v4 (PerkinElmer, Waltham, MA, USA), using 20 ng of input RNA per sample, or the maximum available amount when 20 ng was not achievable. The standard library preparation protocol was followed, with the modification that the tRNA/YRNA blocker solution was omitted to enable capture of tRNAs and tRFs. The completed libraries were pooled and then evaluated using High Sensitivity DNA ScreenTape D1000 (Agilent). 1nM library was sequenced using the NextSeq 1000 platform (Illumina) at single-end 80 bp read setting.

### Bioinformatics analysis of tRNA-derived fragments

tRFs were identified and quantified from small RNA sequencing data using the tRAX software [22], which is designed for comprehensive analysis of tRNA-derived small RNAs (https://github.com/UCSC-LoweLab/tRAX, accessed in 2025). Unlike generic RNA sequencing pipelines, tRAX performs optimized mapping of sequencing reads to both mature and precursor tRNA genes, while accounting for tRNA-specific modifications and isodecoder/isoform sequence variations. This enables precise annotation of cleavage positions and, therefore, accurate classification of tRF subtypes. tRAX was run using default parameters unless otherwise specified, following the recommended pipeline settings. Raw sequencing data underwent adapter removal using TrimGalore tool, and quality control was assessed using FastQC (v0.10.1). Trimmed reads were then processed in tRAX, where they were aligned to *Homo sapiens* reference genome assembly (hg19) using the Bowtie2 aligner. This alignment step was followed by abundance estimation and annotation of tRFs. Importantly, tRAX distinguishes between different classes of tRFs, including: (i) 5′-tRFs, generated from the 5′-end of mature tRNAs, (ii) 3′-tRFs, generated from the 3′ end of mature tRNAs, and (iii) internal tRFs, originating from internal cleavage sites within the tRNA body. Read mapping and classification were performed using the tRAX pipeline assigns each tRF to one of four specificity categories based on sequence uniqueness. Because tRNA genes exhibit high sequence similarity, multi-mapping reads were retained and handled according to the tRAX assignment rules, which collapse reads at the appropriate tRNA isotype, family or fragment level to ensure robust quantification. Reads that map to multiple tRNAs carrying the same amino acid are classified as isotype-specific, while those mapping uniquely to a single anticodon variant are designated isodecoder-specific. Fragments that align exclusively to one genomic tRNA transcript are considered transcript-specific, representing the highest level of mapping precision. Reads that cannot be confidently assigned to any amino acid class are labeled not amino-specific. These specificity classes are represented by distinct colors in the tRF coverage plots, allowing visualization of mapping confidence across tRNA positions. This approach enables discrimination between broadly regulated tRF families and uniquely derived fragments that may serve as precise molecular signatures or biomarkers.

tRF counts were summarized at the fragment level and used as input for DESeq2 differential expression analysis used as the primary statistical framework. DESeq2 models count data using a negative binomial generalized linear model, providing robust inference for high-dimensional sequencing datasets. To account for differences in sequencing depth and RNA composition, counts were normalized using the relative log expression (RLE) size-factor method implemented in DESeq2. Although EB and UF-EV samples were collected from the same general study cohort, a fully matched paired design was not available across all individuals. Therefore, differential expression was performed using an unpaired group comparison framework, without incorporating subject-level blocking factors. Statistical significance was assessed using the Benjamini–Hochberg false discovery rate (FDR) procedure to control for multiple testing, with adjusted p-values < 0.05 considered significant. Differential expression was reported as log2 fold changes (log2FC) with corresponding uncertainty estimates provided by DESeq2, and tRFs with a |log2FC| ≥1 and p adj < 0.05 were defined as significantly differentially expressed tRFs. tRF/tDR (transfer derived RNA) identifiers were assigned using the tRAX database nomenclature (tDRnamer) [23]. When a given fragment/sequence corresponded to multiple valid tDRnamer identifiers (e.g., due to mapping to more than one parental tRNA locus), these were reported as synonymous names and listed together in the results tables.

In addition to the primary DESeq2 framework, secondary statistical analyses were conducted to evaluate phase-associated differences in tRF expression measures across menstrual cycle stages. Specifically, one-way analysis of variance (ANOVA) was applied, followed by Tukey’s honestly significant difference (HSD) post hoc test for multiple pairwise comparisons when appropriate. Each menstrual cycle phase included five to seven independent biological replicates.

## Results

To elucidate the dynamic expression patterns of tRFs across the endometrial cycle, we conducted small RNA sequencing on both EB and UF-EV samples collected at consecutive phases of the menstrual cycle – MP, ES, MS, and LS. Comprehensive small RNA expression profiles were subsequently generated for each sample type to define the distribution of tRFs, assess their fragment-class composition and evaluate phase and compartment-specific differences.

### Dynamics and diversity of tRFs across the menstrual cycle in endometrial biopsies and uterine fluid-derived extracellular vesicles

**Fig. 1:**
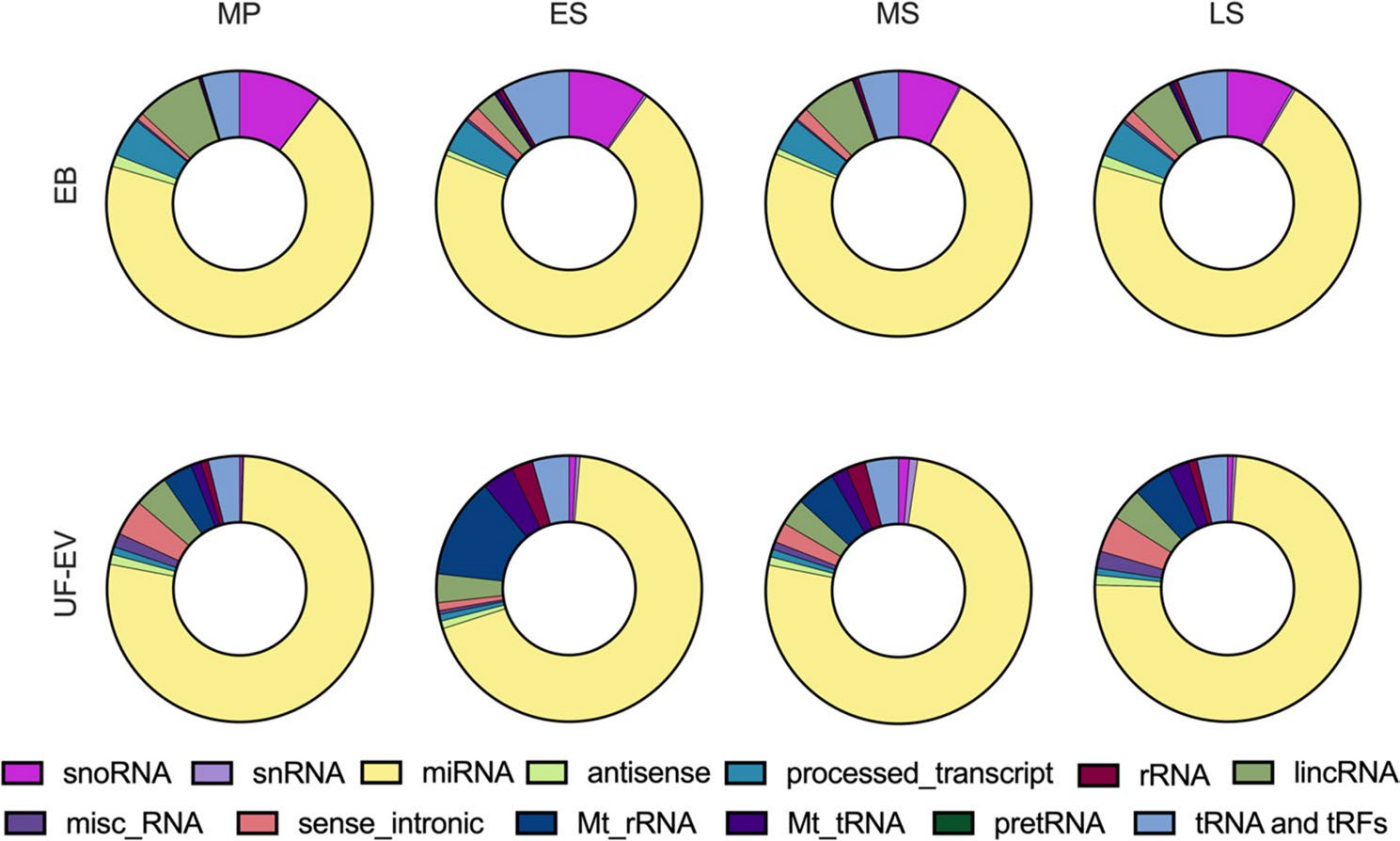
Distribution of small RNA types across the menstrual cycle phases in endometrial biopsies (EB) and uterine fluid-derived extracellular vesicle (UF-EV) samples from healthy women. Samples were collected during the mid-proliferative (MP), early secretory (ES), mid-secretory (MS) and late secretory (LS) phases. For each phase, small RNA category abundances were calculated by summing normalized read counts across all biological replicates (EB: *n* = 5–7; UF-EVs: *n* = 6) and are shown as relative proportions of total mapped small RNA reads. The one-way ANOVA did not detect statistically significant differences at the broad small-RNA class/type level between the menstrual cycle phases in EB and UF-EVs. Small RNA categories are color-coded

Analysis of the small RNA profiles revealed that both EB and UF-EVs were consistently dominated by microRNAs (miRNAs), which accounted for 69–77% of the total small RNA population across all phases (Fig. 1, Supplementary Table 1). Together, tRNAs and tRFs constituted 5–8% of all small RNA species in EB, reaching their highest abundance during the MS phase (8%). In UF-EVs, their abundance remained relatively stable at approximately 4% throughout the menstrual cycle.

**Fig. 2:**
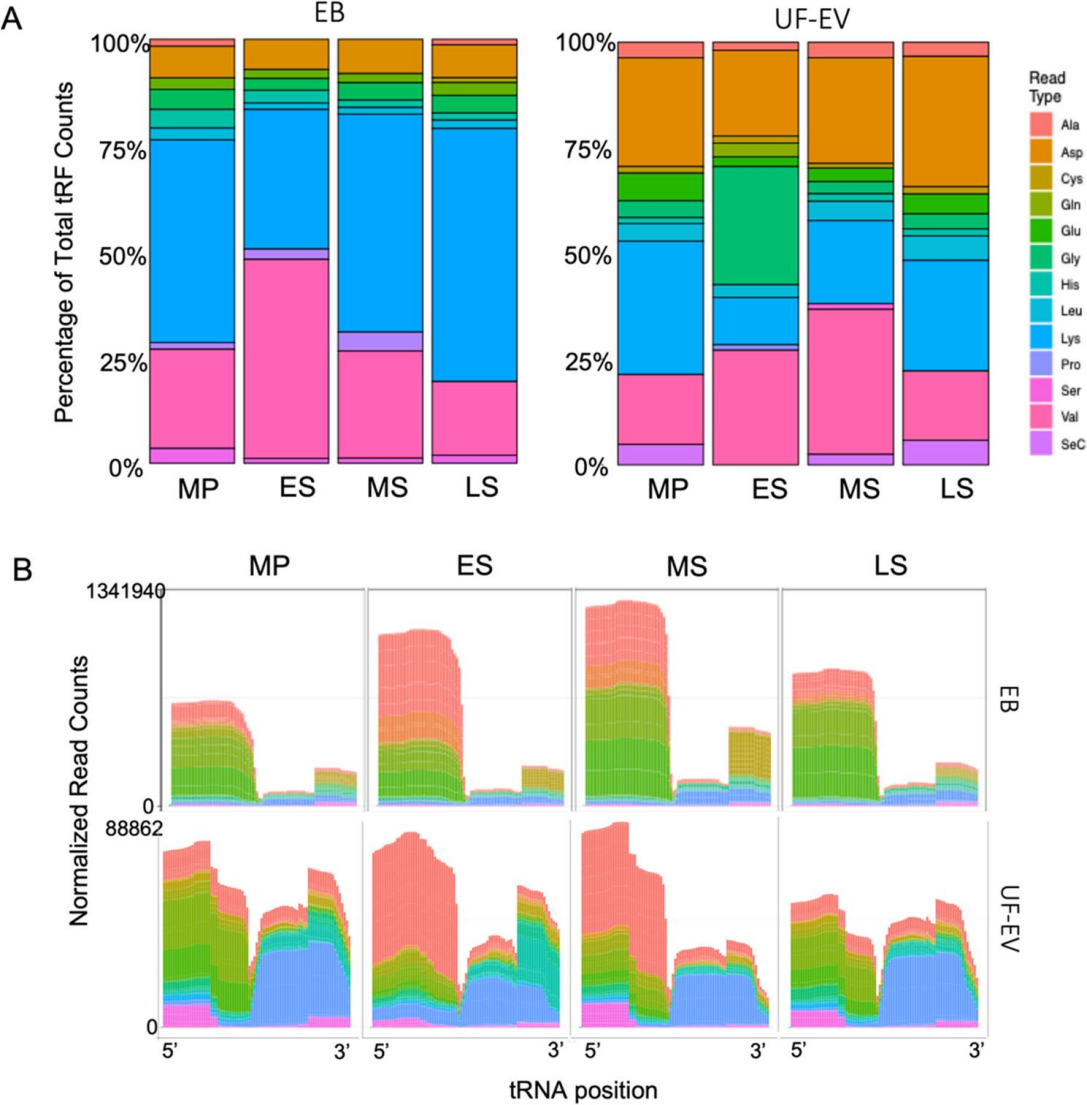
Distribution of tRNA-derived fragments (tRFs) across phases of the menstrual cycle. **A**. Relative abundances of tRFs derived from individual tRNA isotypes detected in endometrial biopsies (EB) and uterine fluid–derived extracellular vesicles (UF-EVs) collected during the mid-proliferative (MP), early secretory (ES), mid-secretory (MS), and late secretory (LS) phases. For each phase, isotype abundances were calculated by summing normalized tRF counts across all biological replicates and are shown as percentages of total mapped tRF reads. **B**. Corresponding normalized read coverage profiles of tRF fragment classes mapped along the 5′–3′ orientation of the parental tRNA. Colors denote fragments derived from specific tRNA isotypes, as indicated in the legend

tRFs were detected from all 21 cytosolic tRNA isotypes, including selenocysteine tRNAs (Fig. 2A, Supplementary Table 2). The expression levels of tRFs varied significantly among tRNA isotypes and exhibited dynamic fluctuations throughout the menstrual cycle. In EB, tRNA-Lysine (Lys) and tRNA-Valine (Val) were the predominant sources of tRFs. Across the menstrual cycle, tRFs derived from tRNA-Lys showed higher expression during the MS and LS phases (49% and 57% of total tRFs, respectively), while tRFs originating from tRNA-Val peaked in the ES phase (45%).

In UF-EVs, the majority of tRFs were derived from tRNA-Lys, tRNA-Val, and tRNA-Aspartic acid (Asp). tRNA-Lys–derived tRFs showed the highest expression in the MP phase (30%), whereas those from tRNA-Val reached their maximum in MS (33%). Unlike EB, tRFs derived from tRNA-Asp were represented across all phases in UF-EVs, ranging from 19% to 29%, tRFs originating from tRNA-Glycine (Gly) displayed a distinct expression peak during the ES phase (26%) (Fig. 2A, Supplementary Table 2).

Overall, tRFs derived from tRNA-Lys and tRNA-Val dominated in EB, whereas UF-EVs exhibited greater tRF diversity, mainly involving tRNA-Lys, tRNA-Val, tRNA-Asp, and tRNA-Gly, reflecting both tissue and phase-specific expression patterns.

The distribution of tRF types across the endometrial cycle in both EB and UF-EVs is presented in Fig. 2B. Importantly, the presented sequencing data consistently revealed a broad spectrum of fragment classes across all sample types and cycle stages, including 5′-, 3′-, and internal tRFs, as reflected by the normalized read coverage profiles mapped along the parental tRNA structure (Fig. 2B). The detection of multiple fragment populations in both EB and UF-EVs suggests that our workflow was able to capture diverse tRF species, rather than being strongly limited to a single subtype. In EB. 5′-tRFs were the predominant tRF category, with dynamic changes in their relative abundance across the phases of the cycle (Supplementary Table 3). While 5′-tRFs remain abundant in UF-EVs, the relative proportion of other tRFs, such as 3′-tRFs and internal tRFs, is higher compared to the EB, suggesting selective packaging or stability of tRF types in UF-EVs (Supplementary Table 4).

### Cycle-dependent modulation of tRNA-derived fragments during secretory phases in endometrial compartments

**Fig. 3:**
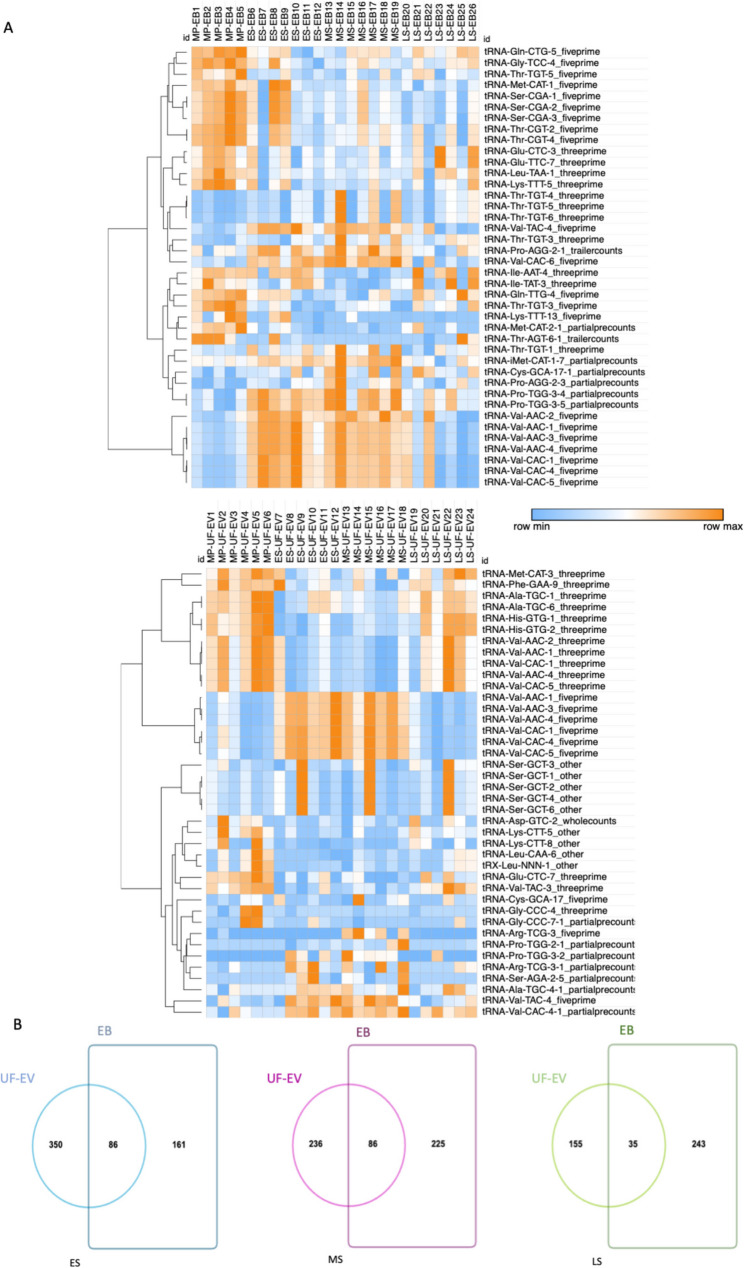
Abundance of tRNA-derived fragment (tRF) types across the menstrual cycle in endometrial biopsy (EB) and uterine fluid extracellular vesicles (UF-EVs). **A**. Heatmap of tRF expression levels in EB and UF-EVs, shown as read counts normalized (log₂) to the total mapped small RNA reads per sample. Hierarchical clustering was performed using Euclidean distance. **B**. Differentially expressed tRFs shared between EB and UF-EVs in the early secretory (ES), mid-secretory (MS), and late secretory (LS) phases compared with the mid-proliferative (MP) phase

Heatmap visualization of normalized tRF counts (Fig. 3A, Supplementary Tables 5 and 6) demonstrated clear phase-dependent variation across the menstrual cycle. The most pronounced changes were observed among 5′-tRFs originating from tRNA-Val (especially from tRNA-Val-AAC and tRNA-Val-CAC isotypes), which exhibited strong enrichment during the ES and MS phases compared with the MP and LS phases. This pattern was consistent in both EB and UF-EVs, suggesting coordinated intracellular accumulation and selective export of tRNA-Val-derived tRFs during the implantation window. In contrast, most fragments derived from tRNA-Thr, tRNA-Ser, and other tRFs showed minimal or no significant fluctuation across the cycle. The secretory-phase enrichment of tRNA-Val-derived tRFs therefore represents the most prominent regulated feature in the dataset, potentially linked to the establishment of endometrial receptivity and EV-mediated signaling. The selective enrichment of specific 5′-tRFs in UF-EVs compared to tissue suggests dynamic tRF sorting or secretion mechanisms across the cycle with notable timing differences in the abundance of the tRF types (Fig. 3B). Collectively, these data indicate dynamic and coordinated regulation of specific tRF populations during endometrial maturation, with secretory-phase enrichment patterns supporting a potential role for tRFs in extracellular communication and endometrial receptivity.

### Phase and compartment-specific upregulation of tRNA-Val 5′-tRFs across the menstrual cycle

**Fig. 4:**
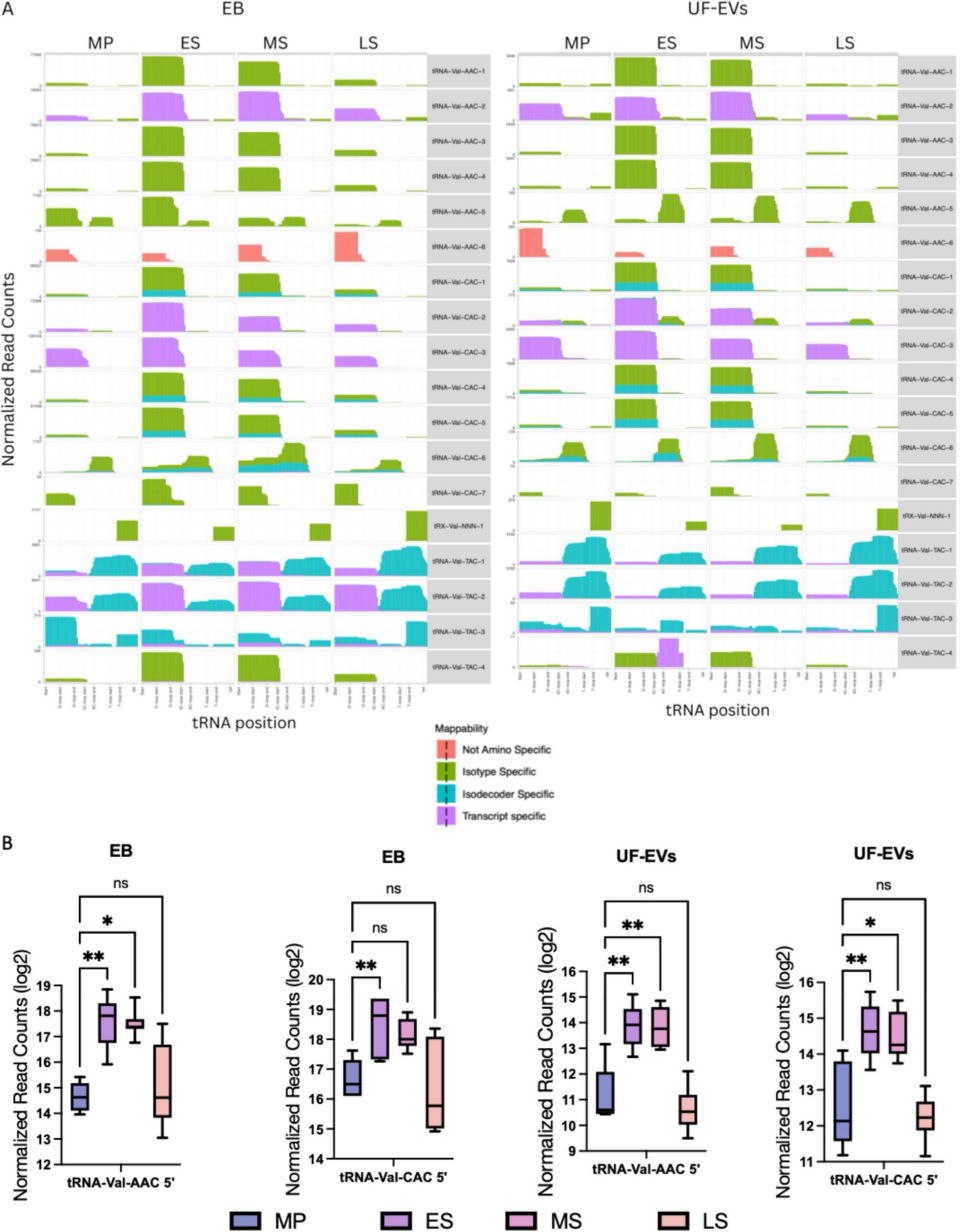
Menstrual cycle-dependent distribution of tRNA-Val-derived fragment (tRF) classes in endometrial biopsy (EB) and uterine fluid extracellular vesicles (UF-EVs) **A**. Positional tRF profiles across the tRNA-Val reference shown as normalized read counts plotted by tRNA position (5′→3′) in the menstrual cycle phase (MP, ES, MS, LS) for EB and UF-EVs. Colored tracks denote the major tRF classes (as indicated in the key) **B**. Fragment-level abundance of tRNA-Val–derived tRFs shown as normalized read counts (log2) for each phase and compartment. The box indicates the data distribution, the line inside the box represents the median, and whiskers extend from the minimum to maximum values. Read counts were normalized to the total mapped small RNA reads per sample. Statistical significance was determined by one-way ANOVA followed by Tukey’s HSD post hoc test. Asterisks indicate statistically significant differences compared to MP (* *p* < 0.05; ** *p* < 0.01)

tRNA-Val-derived fragments displayed distinct menstrual cycle-dependent dynamics, with the highest levels detected during the ES and MS phases in both EB and UF-EVs (Fig. 4A and B). At the fragment level (Table 1), multiple tRNA-Val isodecoder-derived 5′-tRFs were strongly upregulated in ES vs. MP in EB and largely remained elevated in MS vs. MP, indicating sustained secretory-phase induction. UF-EVs broadly mirrored these increases, particularly for AAC- and CAC-derived fragments, although some isodecoder specificity was observed (e.g., tRNA-Val-AAC-2 significant in EB only; tRNA-Val-CAC-6 strongly induced in EB, especially in MS, but non-significant in UF-EVs).

**Table 1:** Fragment-level differential expression of 5′ tRNA-derived fragments (5′-tRFs) originating from tRNA-Val in endometrial biopsy (EB) and uterine fluid extracellular vesicles (UF-EVs) across menstrual-cycle phases. In table, comparisons of early secretory (ES) and mid-secretory (MS) phases versus mid-proliferative (MP) phase for EB and UF-EVs are shown. Fragments were considered differentially expressed at |log2FC|≥1 and padj < 0.05; ns indicates not significant (did not meet the significance threshold)

5’-tRF sources	EB				UF-EVs			
	ES vs MP		MS vs MP		ES vs MP		MS vs MP	
	log2FC	p-adj	log2FC	p-adj	log2FC	p-adj	log2FC	p-adj
tRNA-Val-AAC-2	1.95	1.44E-04	2.00	9.75E-05	ns	ns	ns	ns
tRNA-Val-AAC-3	2.78	6.32E-08	2.51	1.03E-06	3.17	1.16E-10	3.05	5.31E-10
tRNA-Val-AAC-4	2.78	6.39E-08	2.51	1.05E-06	3.18	8.99E-11	3.06	4.16E-10
tRNA-Val-CAC-1	2.81	9.63E-08	2.50	2.08E-06	2.72	1.73E-07	2.61	5.57E-07
tRNA-Val-CAC-2	2.45	2.95E-05	1.66	4.50E-03	2.30	4.68E-05	1.65	3.41E-03
tRNA-Val-CAC-4	2.81	9.63E-08	2.50	2.08E-06	2.72	1.74E-07	2.61	5.60E-07
tRNA-Val-CAC-5	2.80	1.19E-07	2.45	3.63E-06	3.02	4.15E-10	2.90	1.92E-09
tRNA-Val-CAC-6	2.88	1.26E-06	3.78	2.01E-10	ns	ns	ns	ns
tRNA-Val-TAC-4	2.56	5.88E-07	2.44	1.98E-06	2.54	1.21E-04	2.54	1.01E-04

Sequence-level analysis (Table 2 - tRAX/tDRnamer nomenclature with synonymous identifiers grouped and Supplementary Tables 7 - tDR sequences) resolved the secretory-phase response into discrete tRF species and highlighted compartment- and length-specific patterns. In EB, tRNA-Val-CAC–derived sequences dominated, including longer 53–55 nt species that were significantly upregulated in ES and showed stronger induction in MS, consistent with sustained secretory-phase accumulation. EB also exhibited significant increase in shorter 31–36 nt sequence groups mapping to multiple tRNA-Val-CAC and tRNA-Val-AAC isodecoders, indicating coordinated production of specific cleavage products. In contrast, UF-EVs showed no significant changes for the longer 53–55 nt species but preferentially displayed an increase in shorter classes (31–34 nt). Overall, UF-EVs largely recapitulated the endometrial sequence signature while showing selective enrichment of specific short tRF species, consistent with non-random export and/or differential extracellular stability. Together, these results indicate robust secretory-phase upregulation of tRNA-Val 5′-tRFs in endometrium, with UF-EVs largely recapitulating the tissue signature while showing selective enrichment of specific sequence/length classes.

**Table 2:** Sequence-level differential expression of 5′ tRNA-derived fragments (5′-tRFs) originating from tRNA-Val in endometrial biopsy (EB) and uterine fluid extracellular vesicles (UF-EVs) across menstrual-cycle phases. In table, comparisons of early secretory (ES) and mid-secretory (MS) phases versus mid-proliferative (MP) phase for EB and UF-EVs are shown. tRF/tDR names follow the tRAX database nomenclature (tDRnamer); where applicable, synonymous names are listed together (i.e., multiple tRAX/tDRnamer identifiers corresponding to the same sequence are shown in the same row/cell). Fragments were considered differentially expressed at |log2FC|≥1 and padj < 0.05; ns indicates not significant (did not meet the significance threshold)

tDR name	EB				UF-EVs			
	ES vs MP		MS vs MP		ES vs MP		MS vs MP	
	log2FC	P-adj	log2FC	P-adj	log2FC	P-adj	log2FC	P-adj
tDR-1:55-Val-CAC-1	3.03	4.99E-02	3.55	6.80E-03	ns	ns	ns	ns
tDR-1:31-Val-CAC-1 tDR-1:31-Val-CAC-4 tDR-1:31-Val-CAC-5 tDR-1:31-Val-AAC-1 tDR-1:31-Val-AAC-3	2.77	1.05E-02	3.38	5.00E-04	ns	ns	2.32	0.0396
tDR-1:32-Val-CAC-1 tDR-1:32-Val-CAC-4 tDR-1:32-Val-CAC-5 tDR-1:32-Val-AAC-1 tDR-1:32-Val-AAC-3	2.98	1.90E-03	ns	ns	2.51	7.00E-04	2.40	1.20E-03
tDR-1:33-Val-CAC-1 tDR-1:33-Val-CAC-4 tDR-1:33-Val-CAC-5 tDR-1:33-Val-AAC-1 tDR-1:33-Val-AAC-3 tDR-1:33-Val-AAC-4	2.84	4.00E-03	1.88	3.65E-02	2.08	2.50E-03	1.59	4.57E-02
tDR-1:34-Val-CAC-1 tDR-1:34-Val-CAC-4 tDR-1:34-Val-CAC-5	ns	ns	ns	ns	2.22	2.20E-03	1.88	2.91E-02
tDR-1:35-Val-CAC-1 tDR-1:35-Val-CAC-4 tDR-1:35-Val-CAC-5	2.82	4.01E-02	3.78	1.70E-03	ns	ns	ns	ns
tDR-1:36-Val-CAC-1 tDR-1:36-Val-CAC-4 tDR-1:36-Val-CAC-5	3.95	3.74E-02	3.63	2.84E-02	ns	ns	ns	ns
tDR-1:34-Val-AAC-1 tDR-1:34-Val-AAC-3 tDR-1:34-Val-AAC-4	3.56	1.05E-02	3.78	1.70E-03	ns	ns	ns	ns
tDR-1:32-Val-CAC-2	2.92	1.08E-02	2.28	3.23E-02	ns	ns	ns	ns
tDR-1:36-Val-CAC-2	2.79	4.03E-02	ns	ns	ns	ns	ns	ns

## Discussion

This study provides the first comprehensive analysis of tRFs in human EB and UF-EVs across consecutive phases of the menstrual cycle in healthy, fertile women. Our findings demonstrate that tRFs are not only abundant but also subject to dynamic, phase-specific regulation, underscoring their potential importance in endometrial physiology and embryo-maternal communication. This pattern resembles the regulatory roles of other small RNAs in implantation. For example, miRNAs are well-known to regulate the embryo-endometrium signaling network during implantation [24–29], and uterine EVs loaded with regulatory RNAs directly modulate the implantation interface [1, 20, 30, 31]. By analogy, the cyclical shifts in endometrial tRF levels are likely driven by hormonally mediated remodeling events. Notably, the pronounced increase in tRF abundance during the secretory phase suggests that progesterone-induced changes in endometrial cells may promote selective tRNA cleavage or stabilization, which could facilitate the extensive cellular reprogramming required for decidualization, endometrial receptivity, and successful embryo implantation. However, the precise mechanisms by which progesterone may influence tRF generation remain to be elucidated.

Previous studies have revealed that tRFs are not only abundant but also dynamically regulated in the male reproductive tissues of mice [10, 12] and humans [13, 14], with profiles reflecting tissue-specific and developmental cues. Extending these findings to the female reproductive tract, we demonstrate that tRFs are likewise abundant in the endometrium and UF-EVs, exhibiting distinct cycle-dependent expression patterns. Specifically, endometrial tRF populations fluctuate markedly between menstrual phases, with particular tRF species enriched during the secretory phase critical for embryo implantation. These observations suggest that although tRFs have well-established roles in male reproduction, they are also dynamically regulated in the female reproductive tract, correlating with key physiological transitions.

In EB, we observed a predominance of 5′-tRFs, which is consistent with reports that 5′-tRFs represent the major class of tRFs in diverse human tissues. However, tRF profiling using conventional small RNA-seq is subject to library preparation biases, as extensive tRNA nucleotide modifications and heterogeneous fragment end chemistries can affect adapter ligation and reverse transcription efficiency [32, 33]. As a result, certain tRF subtypes may be preferentially captured, potentially contributing to the apparent enrichment of 5′-tRFs in our dataset. The detection of multiple fragment populations in both EB and UF-EVs suggests that the workflow was not strongly restricted to a single subtype and supports that it captured diverse tRF species throughout the endometrial phases. The library preparation protocol used here has also been successfully applied for miRNA identification in this biological system [20], enabling parallel profiling of miRNAs and tRFs and supporting integrated interpretation of these small RNA populations in the endometrial environment. Nevertheless, compartment-specific RNA modification patterns may still influence comparisons between endometrial biopsies and uterine fluid EVs, and cycle-associated differences should therefore be interpreted cautiously.

Although the precise functions of 5′-tRFs in the human endometrium remain incompletely understood, previous studies suggest that these fragments may influence gene expression through several interconnected pathways. For example, 5′-tRFs have been proposed to silence target mRNAs via interactions with untranslated regions, modulate translation initiation through displacement of initiation factors such as YB-1, promote stress granule formation, and possibly contribute indirectly to epigenetic regulation by participating in the generation of other small RNAs [34]. Indeed, broad analyses of human tissues have shown that 5′-tRFs often predominate within tRF populations and exhibit tissue-specific expression patterns linked to pathways governing cell proliferation, differentiation, and stress responses [34, 35]. In studies of ovarian endometriosis, 5′-tRFs were the most dysregulated, underscoring their potential functional importance in reproductive pathologies [15, 36]. Collectively, these observations support the view that 5′-tRFs are not only abundant but also dynamically regulated in the endometrium, with expression profiles that vary with physiological and pathological states.

Recent studies have reported a diverse set of tRFs in the oviduct and endometrial fluids of female mice during the preimplantation period [18]. As noted previously, sperm-derived tRFs can influence gene expression during early embryonic development, thus mediating paternal effects on early embryos [12]. It was further demonstrated that maternal tRFs can affect implantation by modulating the embryo’s metabolic status, being shaped by mice maternal diet [18]. Together with our findings, these reports raise the possibility that tRFs may also contribute to early reproductive events in humans. However, further functional and mechanistic studies will be required to determine whether such roles are conserved and to clarify the biological significance of cycle-dependent tRF dynamics observed in the present study.

In our data, tRFs derived from tRNA-Val showed dynamic regulation in EB and UF-EVs across the menstrual cycle. A recent study by Liu and collaborators [11] revealed that a 5′-tRFs derived from tRNA-Val-CAC is highly enriched in mature mouse sperm and is selectively packaged into epididymosomes - EVs secreted by epididymal epithelial cells and interacting with the RNA-binding protein heterogeneous nuclear ribonucleoprotein A/B (hnRNPAB). This selective sorting mechanism ensures delivery of the tRFs of tRNA-Val-CAC to sperm during post-testicular maturation. When these tRF-Val-CAC fragments were microinjected into mouse oocytes at the time of fertilization, they induced widespread alterations in mRNA processing and alternative splicing within preimplantation embryos. Notably, expression of key splicing regulators such as hnRNPA1, Rbmx, and Srsf10 was affected. These perturbations were associated with delayed embryonic development and reduced progression to the blastocyst stage, indicating that tRF-Val-CAC may influence early developmental processes in this model system. Interpretation of these results should consider the inherent limitations of endometrial research. Phase-specific sample sizes were modest due to the invasive nature of endometrial biopsies and the non-standardized collection of uterine fluid in clinical settings. Moreover, inclusion of endocrine parameters, such as estrogen and progesterone levels, together with histological assessment, may further refine interpretation of tfRNA expression patterns. Additionally, the cross-sectional design captures phase-associated differences between individuals rather than cyclical changes within the same person. Furthermore, we cannot exclude the possibility that some of the tRFs could originate from co-isolated extracellular ribonucleoprotein complexes, rather than the vesicular RNA itself, as the isolated EVs were not subjected to RNase treatment.

Although the previously published work primarily focuses on male gamete maturation and early embryogenesis in mice, it raises the possibility that selective processing and EV-associated transport of tRFs could represent a broader mechanism of RNA-based communication in reproductive biology. In this context, the cyclical modulation and EV enrichment of tRFs from tRNA-Val observed in the human endometrium may suggest a potential involvement in intercellular signaling during endometrial remodeling or embryo-maternal interactions. However, further functional studies will be necessary to determine whether such mechanisms operate in the female reproductive tract and to clarify the biological significance of tRF-Val dynamics in human fertility.

## Conclusion

In summary, our findings identify tRFs as regulated components of the endometrial small-RNA landscape that vary across the menstrual cycle. Alongside miRNAs and other non-coding RNAs, tRFs may contribute to molecular signaling at the embryo–maternal interface. EVs are increasingly recognized as key mediators of implantation-related communication through the transfer of bioactive RNA and protein cargo. In this context, the phase-dependent enrichment of tRFs in uterine fluid EVs suggests that these fragments are selectively represented in the extracellular compartment during the secretory phase. Notably, the pronounced secretory-phase increase of specific tRNA-Val–derived 5′-tRFs supports their potential relevance to uterine receptivity and highlights their promise as candidate biomarkers of endometrial status.

## Supplementary Information

Supplementary Material 1.

Supplementary Material 2.

Supplementary Material 3.

Supplementary Material 4.

Supplementary Material 5.

Supplementary Material 6.

Supplementary Material 7.

## Data Availability

The raw small RNA-seq data have been deposited in NCBI under the accession number [PRJNA1367015](https://www.ncbi.nlm.nih.gov/bioproject/1367015) .

## Abbreviations

EB: Endometrial biopsy
UF: Uterine fluid
UF-EVs: Uterine fluid–derived extracellular vesicles
EVs: Extracellular vesicles
WOI: Window of implantation
LH: Luteinizing hormone
MP: Mid-proliferative phase
ES: Early secretory phase
MS: Mid-secretory phase
LS: Late secretory phase
tRFs: tRNA-derived fragments
tiRNAs: tRNA halves
miRNAs: microRNAs
nt: Nucleotides
i-tRFs: Internal tRNA-derived fragments
tRF-1: tRNA-derived fragments from the 3′ trailer of precursor tRNAs
tRF-2: tRNA-derived fragments spanning the anticodon loop
tRAX: tRNA Analysis Tool
SEC: Size-exclusion chromatography
Lys: Lysine
Val: Valine
Asp: Aspartic acid
Gly: Glycine
Thr: Threonine
Ser: Serine
